# Odour dialects among wild mammals

**DOI:** 10.1038/s41598-017-12706-8

**Published:** 2017-10-19

**Authors:** Eleanor Freya Kean, Michael William Bruford, Isa-Rita M. Russo, Carsten Theodor Müller, Elizabeth Anna Chadwick

**Affiliations:** Cardiff University, School of Bioscience, Museum Avenue, Cardiff, CF10 3AX Wales

## Abstract

Across multiple taxa, population structure and dynamics depend on effective signalling between individuals. Among mammals, chemical communication is arguably the most important sense, underpinning mate choice, parental care, territoriality and even disease transmission. There is a growing body of evidence that odours signal genetic information that may confer considerable benefits including inbreeding avoidance and nepotism. To date, however, there has been no clear evidence that odours encode population-level information in wild mammals. Here we demonstrate for the first time the existence of ‘odour dialects’ in genetically distinct mammalian subpopulations across a large geographical scale. We found that otters, *Lutra lutra*, from across the United Kingdom possess sex and biogeography-specific odours. Subpopulations with the most distinctive odour profiles are also the most genetically diverse but not the most genetically differentiated. Furthermore, geographic distance between individuals does not explain regional odour differences, refuting other potential explanations such as group odour sharing behaviour. Differences in the language of odours between subpopulations have the potential to affect individual interactions, which could impact reproduction and gene-flow.

## Introduction

Chemical communication, arguably the most important mode of mammalian communication^1^, is commonly associated with territorial marking^2^ but it also plays a vital role in mate attraction, mate choice and reproduction^3^, parental care^4^ and even disease transmission^5^. Intraspecific chemical communication therefore affects a plethora of behavioural interactions, and in the context of reproduction is essential for maintenance of animal population structure. As well as signalling key identifiers such as sex and age^6^, there is evidence that differences in odour may allow genetic differences to be detected at a variety of scales. On a taxonomic scale, there are differences in odours between species (e.g. canids^7^) and to some extent subspecies (e.g. beavers^8^), whereas at a much finer scale, there is emerging evidence that within small groups there are odour signals of relatedness and genetic quality, for example, within captive primate groups^9–11^, within beaver families^12^ or between laboratory mice^13^ and more recently in wild fur seals^14^. The communication of genetic similarity through odours on this individual scale has been suggested to function in phenotype matching^12^, mate choice for genetic diversity and MHC genotype^11^, kin recognition and inbreeding avoidance or nepotism^9,10^.

Gene-flow among wild populations is affected by geographic features and for many species individuals that are geographically closer are more likely to be related than those further apart^15^. Consequently, geographical separation of wild animal populations (either through natural processes and barriers, or through anthropogenic fragmentation of habitats) results in reduced gene-flow and thus genetic structuring of populations. It remains unclear, however, whether associations between individual genetic relatedness and odour that are found in laboratory and captive populations are also present at the sub-population level, or in wild vertebrates living across large geographical areas. The relationship between population genetic structure and intraspecific odour communication has thus far received little attention, but recent evidence suggests that geographical separation may drive a divergence in chemical signals^16^. Laboratory based choice experiments provide some evidence that olfactory discrimination could act a mechanism for reproductive isolation and speciation^17^; differences in chemical signals could therefore impede reproduction between sub-populations. In the case of species of conservation concern, such differences could therefore impede species recovery even where, for example, conservation measures reduce habitat fragmentation. At a higher taxonomic level, chemical signals may provide effective barriers to hybridization^18^. Olfactory discrimination is therefore potentially fundamental to adaptation and evolution via the maintenance of species boundaries^18^.

**Figure 1:**
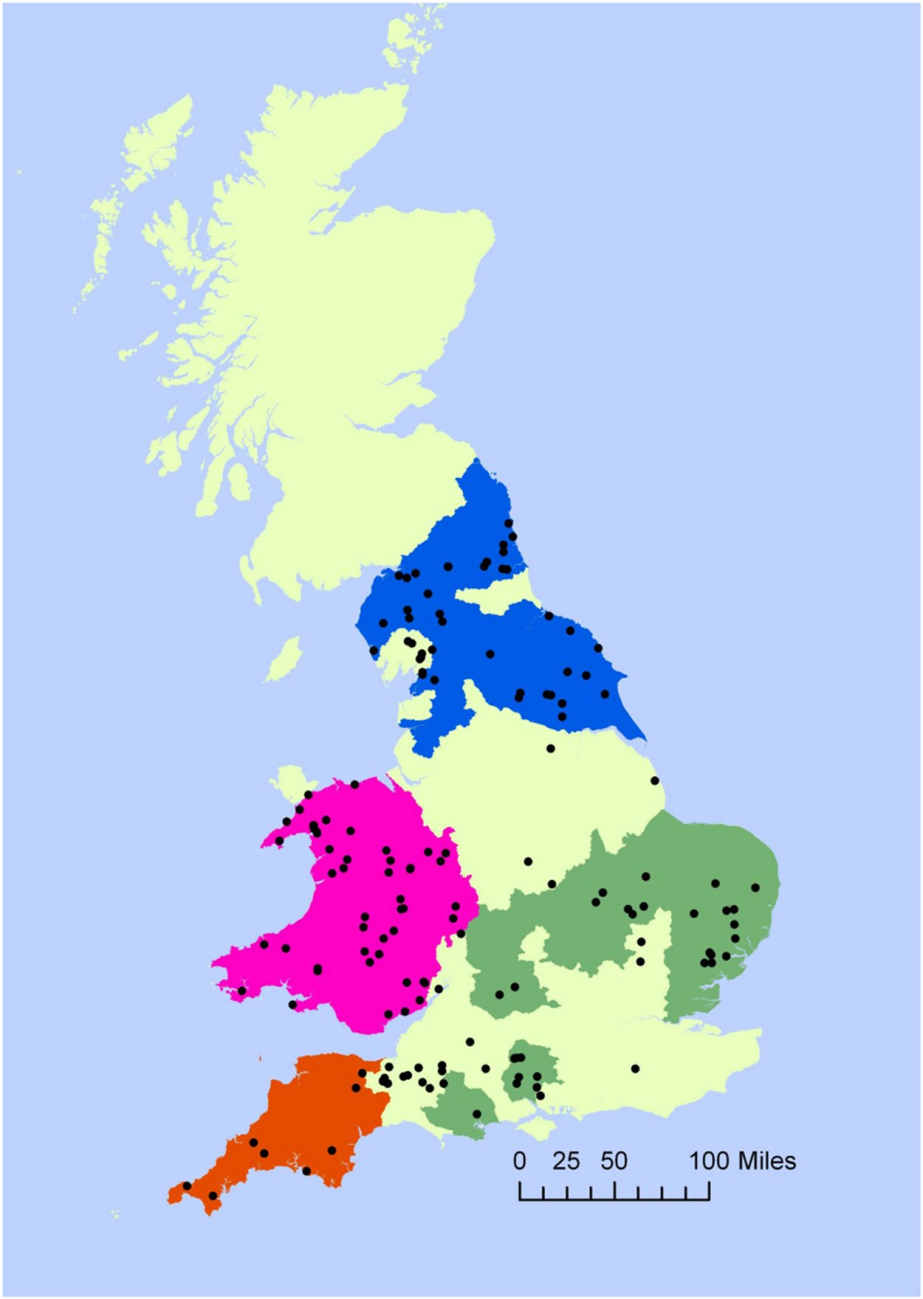
Distribution of otter anal gland samples across four genetically distinct subpopulations in England and Wales. Pink = Wales and Borders (W&B), Red = South-west England (SW), blue = North England (NE), Green = Central England (CE). Samples outside the four subpopulations were excluded from the present analysis. Map created using ArcGIS® software ArcMap™ 10.1 Copyright © Esri www.esri.com. Outline of genetically distinct subpopulations created using data from Hobbs *et al*.^27^.

We investigated the relationship between genetic information and odour profiles in Eurasian otters (*Lutra lutra*) over a large geographic area across England and Wales (maximum distance, 630 km). Otters possess two anal sacs positioned on either side of the rectum with ducts opening just close to the anus. Secretion from these glands is deposited with faeces (known as spraint) in prominent locations in a way that is typical of scent marking^19^. It has been assumed that spraint is used to signal territorial boundaries, usage of food resources, to aid navigation or attract mates^20–25^. More recently it has been shown that the odour of otter anal gland secretions is significantly associated with individual’s age, sex, reproductive status^6^ and individual identity^26^. Genetic sub-structuring in UK otters is known to relate to geographical location, probably as a result of re-colonisation from small and spatially separated remnant populations surviving severe declines in otter numbers across the UK during the 1960s^27^. Based on genetic data, four major subpopulations in the UK have been described that were significantly differentiated from each other: Wales and Borders (W&B), South-west England (SW), North England (NE) and Central England (CE) (Fig. 1), with the greatest degree of differentiation detected between the SW and the W&B regions^27^.

Here, we measured volatile organic compounds (VOCs) from otter anal glands, tested whether these varied spatially and related these results to population genetic differentiation. Otter anal gland secretions were collected from across four distinct subpopulations in the UK^27^, and tested to ascertain whether odour variation was associated with genetic sub-structuring, discriminating statistically whether variation was based on spatial proximity or genetic distance. We hypothesised that (1) odour profiles would differ between genetically distinct subpopulations, (2) groups with the most distinct odours would also exhibit the highest genetic differentiation, and (3) geographical distance between individuals would not on its own explain odour differences between groups.

## Methods

### Samples

Otters found dead in England and Wales were collected (under license from Natural England or Natural Resources Wales) between 2004 and 2009, and stored at −20 °C until defrosted for post mortem examination. Only those showing minimal autolysis were selected for the present study, n = 122 (see Supplementary Table S1 online for sample size in each category), thus controlling for inevitable differences in time since death. Carcasses were collected throughout the year, but with low levels of mortality during the summer months. Otter sex, age-class (juvenile, sub-adult, adult) and adult female reproductive status (pregnant, lactating, quiescent) were determined. The otters were assigned to one of four genetically distinct subpopulations in England and Wales according to the location where they were found dead, using the boundaries among genetically distinct subpopulations established by Hobbs *et al*.^27^ (Fig. 1), these being North England, Central England, Wales and Borders and South-west England. Only female samples were available from the South-west subpopulation. Anal glands were removed from the otter carcasses during post mortem examination, foil wrapped and stored in ziplock bags at −20 °C for a maximum of five years, prior to analysis glands were defrosted at approximately 4 °C. The entire contents of both glands were expressed manually into one 10 ml SPME vial (Supelco) and left to equilibrate at room temperature for one hour.

### Odour analyses

Volatile organic compounds (VOCs) emitted from the secretion of the anal glands were analysed using solid-phase microextraction (SPME) and gas chromatography mass spectrometry (GCMS) (see Kean *et al*. 2011^6^). After every 4–6 samples, 0.2 µl of an external hydrocarbon standard (MA EPH Aliphatic Hydrocarbon Standard [Restek] diluted 1:50 with n-hexane) was injected using an automatic liquid injector to check the performance of the GC-MS and for calculation of retention indices. This allowed standardisation of retention times. Compounds were provisionally identified (minimum match factor between the deconvoluted component and the library spectra of 80%) and quantified using Automated Mass Spectral Deconvolution and Identification System (AMDIS) version 2.65 and the NIST Mass Spectral Library Version 2.0 (2005). Both mass spectral data and retention indices were used by AMDIS in identification. Peaks with a retention time below 2 min were not included in the analysis because peaks with retention times close to the hold-up time of the system are not measurable with sufficient accuracy. Since an internal standard was not used, absolute values could not be measured; instead the relative contribution of each peak to the overall scent profile was calculated, that is, the data were normalised. Data were also standardised across compounds to ensure compounds were given equal weight in the analysis, irrespective of their size. Zero values were replaced with half the value of the lowest intensity compound measured in the entire data set.

### Statistical analyses

Differences in odour profiles between the four subpopulations were tested using Permutational Multivariate Analysis of Variance (PerMANOVA), with a Euclidean distance matrix and 999 permutations. PerMANOVA was used to investigate whether subpopulation explained any variation in the odour data (432 VOCs) while controlling for sex and age (modelled on three of the four regions, with the SW subpopulation excluded because data deficiency for males precluded the interaction term). PerMANOVAs were performed using the function ‘adonis’ in the vegan package^28^ in R (version 3.3.3)^29^. Post-hoc tests do not exist for the ‘adonis’ function, so individual models were performed on male and female data. Female reproductive status was controlled for in the female only models. Canonical analysis of principal coordinates using ‘ordiplot’ in the vegan^28^ and BiodiversityR packages^30^, was used to create an ordination plot to visualise the VOC profile differences between the sub-populations.

Pairwise F_ST_ values among subpopulations were previously published^27^. In addition, we calculated pairwise F_ST_ values within each subpopulation between males and females in ARLEQUIN 3.5^31^. Statistical significance was tested with 10,000 permutations as implemented in ARLEQUIN.

To test whether subpopulation odour differences were based purely on geographical distances between samples, a Mantel test was performed using the vegan package^28^ in R (version 3.3.3)^29^. This tested for correlation between two dissimilarity matrices: geographical location where the otter was found (x and y coordinates) and all odour data (432 VOCs). The Mantel test was based on Pearson product moment correlation with 999 permutations.

### Data availability

The datasets generated during and/or analysed during the current study are available from the corresponding author on reasonable request.

## Results

**Figure 2:**
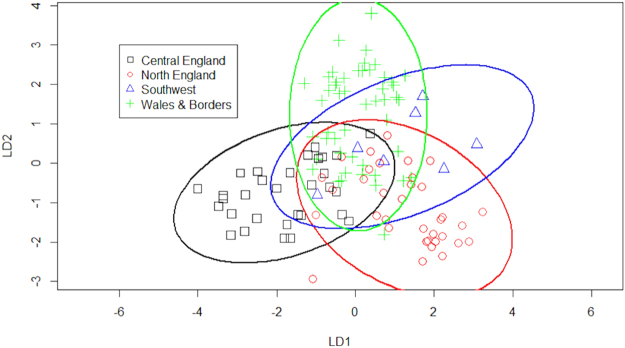
Partial discrimination between four genetically distinct sup-populations based on otter odour. Ordination plot of linear discriminants (LDs) 1 and 2 of the total otter VOCs. Ellipses represent the 95% confidence interval.

The VOCs measured from otter anal gland secretions have already been described^6^ and comprise a complex mixture of organic acids, their esters, alkanes, alkanols, aldehydes and ketones, aromatic compounds, furanes, and nitrogen and sulphur containing compounds. Marked differences in anal gland VOCs of otters were found between the four genetically distinct subpopulations (after controlling for differences with sex, age and female reproductive status; Table 1). Ordination showed partial discrimination between these four subpopulations (Fig. 2). Differences in otter odours between genetic regions were sex dependent (excluding the SW, Table 1). Subsequent separate models for females only (including the SW) and for males only (excluding SW) both revealed significant odour differences between subpopulations (females, *F*_3,73_ = 1.51, *R* ^2^ = 0.05, *p* = 0.04; males, *F*_2,42_ = 1.37, *R* ^2^ = 0.06, *p* = 0.01).

**Table 1:** Otter odour (VOCs) in relation to subpopulation, sex and age tested using PerMANOVA.

	df	SS	MS	Pseudo- *F*	*R* ^*2*^	P
**Subpopulation**	2	972	485.99	1.22	0.02	**0**.049*
**Sex**	1	1326	1325.95	3.32	0.03	**0**.001***
**Age**	2	2497	1248.26	3.12	0.05	**0**.001***
**Subpopulation × Sex**	2	1093	546.3	1.37	0.02	**0**.017*
Subpopulation × Age	4	1732	433.07	1.08	0.04	0.244
**Sex × Age**	2	1188	593.79	1.49	0.02	**0**.018*
Subpopulation × Sex × Age	2	770	385.21	0.96	0.02	0.57
Residuals	99	39557	399.56		0.81	
Total	114	49134			1	

df = degrees of freedom, SS = sum of squares, MS = mean squares. Bold font indicates statistical significance (p < 0.05) for both odour and genetic analyses. *p < 0.05, **p < 0.01, ***p < 0.001. Modelled on three of the four regions, with SW subpopulation excluded because data deficiency for males precluded the interaction term. Female reproductive status was included in a female only data model, the genetic region: reproductive status interaction was not significant so the results are not presented.

Five of nine pairwise *post hoc* comparisons were statistically significant (Table 2) providing strong support for an association between odour, subpopulation and sex. *Post hoc* tests indicated that females originating from the NE subpopulation had significantly different odours to females from all other subpopulations (Table 2). Additionally, males from CE had significantly different odours to males from W&B, and NE subpopulations (Table 2). In contrast, there was no significant differentiation at the genetic level for males and females (not shown), i.e. sex specific subpopulation odour differences are not explained by sex differences in the genetic structure of the UK otter population. We hypothesised that the groups with the most distinct odours (those significantly differentiated, Table 1) would be the same groups with highest genetic differentiation (i.e. SW and W&B^27^), but this was not the case. All pairwise *F*_ST_ values were significant (Table 2) therefore both males and females contributed equally to the genetic differentiation between subpopulations.

**Table 2:** Otter odour (PerMANOVA analysis) and genetic (*F*_ST_) pairwise comparisons between subpopulations, for males and females separately.

Pairwise comparison of subpopulations	Odour analyses					Genetic analyses
	Sex	df	Pseudo-*F*	*R* ^*2*^	*p*	*F*_ST_
NE – CE	Females	1, 38	1.65	0.04	**0**.007**	**0**.10***
	Males	1, 24	1.49	0.06	**0**.018*	**0**.10***
NE – W&B	Females	1, 46	1.73	0.03	**0**.004**	**0**.19***
	Males	1, 32	1.26	0.04	0.112	0.18***
CE – W&B	Females	1, 46	1.08	0.02	0.251	0.23***
	Males	1, 28	1.49	0.05	**0**.029*	**0**.22***
SW – NE	Females	1, 25	1.84	0.06	**0**.030*	**0**.20***
SW – CE	Females	1, 25	1.18	0.04	0.220	0.23***
SW – W&B	Females	1, 33	1.13	0.03	0.252	0.28***

The lack of male samples from South-west England prevented pairwise comparisons. W&B = Wales and Borders, SW = South-west England, NE = North England, CE = Central England. Bold font indicates statistical significance (p < 0.05) for both odour and genetic analyses. *p < 0.05, **p < 0.01, ***p < 0.001.

We found no correlation between odour similarity and spatial distance, either for the dataset as a whole (comparison of distance matrices, *r* = 0.02, *n* = 158, *p* = 0.13), or where sexes were examined separately (adult male samples *r* = 0.07, *n* = 27, *p* = 0.11; adult female samples *r* = −0.04, *n* = 24, *p* = 0.67). Our results show that associations between odour and location are not based simply on geographical distance between individuals, but reflect subpopulation genetic differentiation.

## Discussion

Our analyses support our first hypothesis that odour varies between genetically distinct subpopulations. Previous evidence of odour similarity reflecting genetic similarity has been derived from studies of relatedness at the individual level^9–14^. Evidence at population level is rare, and is often confounded - for example, a study on wild bat colonies showed an association between mitochondrial haplotype and odour similarity, but the association was just as likely to correlate with colony membership as it was to reflect genetic similarity^32^. Our study provides the first evidence of genetic and odour similarity at the genetically identified population level, and is one of very few on free-ranging wild populations.

The distinctiveness of odours detected among males from CE and females from NE may reflect aspects of their genetic history other than differentiation. The CE and NE otter population clusters had a higher number of unique alleles, and higher allelic diversity compared with SW and W&B subpopulations^27^. This higher genetic diversity and private alleles are likely due to reintroductions made with otters originating from outside the introduction area, and possibly including non-native individuals (summarised in Hobbs *et al*.^27^). Higher diversity in NE may also reflect immigration from Scotland, which is reported to have greater genetic diversity than southern UK populations^33^.

Variables other than genetic sub-structuring could account for spatial variation in odours, for example similar diet and habitat could explain an association between odour similarity and spatial proximity. Many group-living species engage in behaviours that promote shared odours to aid group cohesion (e.g. badgers, which simultaneously press together their sub-caudal regions, where glands are located^34^). Studies in which a link between genetic and odour differences are inferred from spatial correlation alone, without explicit testing of genetic differences, may therefore be confounded. The anal gland odour of otters, presented here, showed an association with its location of origin, but only in terms of the genetically distinct subpopulation it belongs to. Geographic distances between individual animals are not always relevant to genetic relatedness, for example where barriers to migration (e.g. large roads) or habitat less favourable to movement restrict breeding between spatially adjacent individuals. By testing for both spatial and genetic differences in odour, we support the growing body of evidence^9–14,17,18,35^, of associations between odour and genetic diversity and differentiation.

Previous research has shown that fragmentation of wild animal populations leads to reduced gene flow and in some cases reduced population viability^36^. Here we provide evidence that genetically distinct subpopulations differ in their odour profile, suggesting that communication between individuals might also be affected by fragmentation. This raises the intriguing possibility that population fragmentation may result in the accumulation of distinct odour profiles in populations, thus adding odour traits to the range of phenotypic characteristics that may diverge under environmental change^37^. To identify the significance of results presented here, investigation into recognition of odour differences and behavioural responses is needed^35^. Should avoidance of, or attraction to, unfamiliar odour profiles occur, then there are broad implications for reproduction and gene flow, and thus the recovery of wild animal populations.

## Electronic supplementary material

Supplementary Table S1.
