# Supplementary material for: Odour dialects among wild mammals
Source: Sci Rep. 2017 Oct 19;7:13593. doi: 10.1038/s41598-017-12706-8 (PMC5648788; doi:10.1038/s41598-017-12706-8)
Supplement: Supplementary file 1 — Supplementary Table S1. [file 41598_2017_12706_MOESM1_ESM.pdf]

Title: Odour dialects among wild mammals

Authors: Kean, E.F., Bruford, M.W., Russo, I.M., Müller, C.T., Chadwick, E.A.

Supplementary Table S1. Number of otters from four genetic subpopulations sampled for anal gland odour analysis

	**Female**			**Male**			**Total**
	Adult	Sub-adult	Juvenile	Adult	Sub-adult	Juvenile	
**Central England**	12	8		6	4	1	31
**North England**	15	5		4	7	4	35
**South-west England**	5	1	1				7
**Wales & Borders**	17	7	6	7	8	4	49
**Total**	49	21	7	17	19	9	122
